# Cardiac conduction abnormalities and congenital immunodeficiency in a child with Kabuki syndrome: Case report

**DOI:** 10.1186/1471-2350-6-28

**Published:** 2005-07-25

**Authors:** Maulik Shah, Brian Bogucki, Melissa Mavers, Daphne E deMello, Alan Knutsen

**Affiliations:** 1 Division of Medical Genetics, Department of Pediatrics, Saint Louis University, 1465 South Grand Blvd., Saint Louis, MO, 63104-1095, USA; 2 Saint Louis University Cancer Center, 3655 Vista Ave., Saint Louis, MO, 63110, USA; 3 Department of Pathology, Saint Louis University, 1402 South Grand Blvd., Saint Louis, MO, 63104, USA; 4 School of Medicine, Saint Louis University, 1402 South Grand Blvd., Saint Louis, MO, 63104, USA; 5 Division of Allergy and Immunology, Department of Pediatrics, Saint Louis University, 1565 South Grand Blvd., Saint Louis, MO, 63104-1095, USA

## Abstract

**Background:**

Since it's recognition in 1981, a more complete phenotype of Kabuki syndrome is becoming evident as additional cases are identified. Congenital heart defects and a number of visceral abnormalities have been added to the typical dysmorphic features originally described.

**Case Report:**

In this report we describe the clinical course of a child diagnosed with Kabuki syndrome based on characteristic clinical, radiological and morphologic features who died of a cardiac arrhythmia at 11-months of age. This infant, however, had abnormal pulmonary architecture and alterations in his cardiac conduction system resulting in episodes of bradycardia and asystole. This child also had an immunological phenotype consistent with common variable immunodeficiency. His clinical course consisted of numerous hospitalizations for recurrent bacterial infections and congenital hypogammaglobulinemia characterized by low serum IgG and IgA but normal IgM levels, and decreased antibody levels to immunizations. T-, B- and NK lymphocyte subpopulations and T-cell function studies were normal.

**Conclusion:**

This child may represent a more severe phenotype of Kabuki syndrome. Recurrent infections in a child should prompt a thorough immunological evaluation. Additionally, electrophysiology testing may be indicated if cardiopulmonary events occur which are not explained by anatomic defects.

## Background

Kabuki syndrome (KS) is a multiple congenital anomalies/mental retardation (MCA/MR) syndrome of unknown cause. It was first described by Niikawa et al. [1] in 1981 and is also known as Niikawa-Kuroki syndrome. In the interim, more than 300 patients of both Asian and non-Asian heritage have been reported[2]. Although a few associated cytogenetic abnormalities have been reported[3], the majority of patients have normal chromosomes and no genetic basis has to date been identified. The inheritance pattern of this disorder has not been established. Most cases are sporadic, but a few families with multiple generations of affected individuals have been reported suggesting autosomal dominant inheritance[4]. Diagnosis is based on characteristic dysmorphic features, visceral abnormalities and clinical features. Common dysmorphic features include arched eyebrows, eversion of the lateral lower eyelid, cleft palate, bifid uvula, and persistent fetal finger tip pads[5]. Visceral abnormalities often include structural heart defects[6] and abdominal wall defects [7]. Other clinical features may include microcephaly and post-natal growth retardation. In addition, recurrent infections, principally otitis media, as well as upper respiratory tract infections and pneumonia occur in patients with KS[8]. However, antibody immune defects have been described in only isolated patients[9].

Structural heart defects are encountered in 32% – 58% of children and no specific congenital heart defect predominates[2]. Although heart defects in the ventricular septum may cause cardiac rhythm disturbances, in our patient, bradyarrythmia progressing to asystole and death was the primary clinical problem resulting from an abnormal conduction system.

## Case presentation

J.R. was a 3210 gram child born to a 21-year-old gravida 1, para 1 mother by vaginal delivery at 36 weeks of gestation. The mother received appropriate prenatal care and the pregnancy was uncomplicated. There was no gestational history of tobacco, alcohol, illicit substance or medication use. A cardiac echogenic focus and possible anatomic heart defect was noted on routine prenatal ultrasound examination and the child was transferred to the neonatal intensive care unit for management at birth. A thoracic echocardiogram showed mitral stenosis, aortic stenosis and coarctation of the aorta. Surgical repair of the coarctation was conducted. A right diaphragmatic eventration was simultaneously repaired. During this initial hospitalization no cardiac arrhythmias were monitored on telemetry. Due to the structural heart defects a chromosome analysis including fluorescence in situ hybridization for both DiGeorge loci was performed and revealed no abnormalities. A diagnosis of Kabuki syndrome was made by two independent geneticists based on phenotypic features. Clinical features in this child consistent with the diagnosis of Kabuki syndrome included arched eyebrows with sparse hair laterally, eversion of the lateral lower eyelid, a cleft palate, bifid uvula, broad nasal root with depressed nasal tip, and persistent fetal finger tip pads with otherwise normal dermatoglyphics. Neuroimaging revealed severe corpus callosum hypoplasia and ophthalmologic examination showed optic nerve atrophy bilaterally. Congenital hypothyroidism was also detected. Further cytogenetic analysis using telomere probes was conducted without detection of abnormalities.

The subsequent hospital course was complicated by numerous infections secondary to hypogammaglobulinemia and he was finally discharged to home at 3 months of age. After one week at home, he was noted to be cyanotic and non-responsive and was re-admitted to the hospital where the patient was considered to be septic and placed on broad spectrum antibiotics.

At 6.5 months of age, he was admitted to the hospital with a urinary tract infection. Sinus bradyarrythmia was recorded associated with hypoxemia with prolonged pauses progressing to asystole. Resuscitation with epinephrine was successful. Bronchoscopy was performed and showed normal airways. A cardiac echocardiogram showed diastolic dysfunction. He was started on supplemental O_2 _at 1/8 L by nasal canula and received furosemide, aldactazide and verapamil. No other episodes of cardiac rhythm disturbance were noted on telemetry during the remaining hospital stay.

At 9 months of age, he was admitted to the hospital in respiratory distress. He was ventilated mechanically and monitored in the ICU. While on cardiac monitoring he developed a tachyarrhythmia without 1:1 conduction then prolongation of the QT interval followed by an idioventricular rhythm which slowed to asystole. External pacing failed. After alternate doses of epinephrine and atropine the patient returned to sinus rhythm. Thoracic echocardiogram showed normal ventricular function with normal velocities across inlet and outlet valves. Troponin levels remained less than 1 and there was no other evidence of ischemia. Because of the previous episodes of bradyarrythmia, a dual chamber epicardial pacemaker was placed without complications. His ventricular and diastolic function normalized and diuretics were discontinued. He remained hemodynamically stable but continued hospitalization was required for various nosocomial infections. The family elected to make an advance directive to prevent further resuscitative efforts. Approximately one month later during hospitalization for respiratory bronchiolitis, he had another episode of bradyarrythmia which progressed to asystole and death. The pacemaker was queried and found to have functioned within normal parameters. The family consented for a limited autopsy.

### Cardiovascular autopsy findings

**Figure 1:**
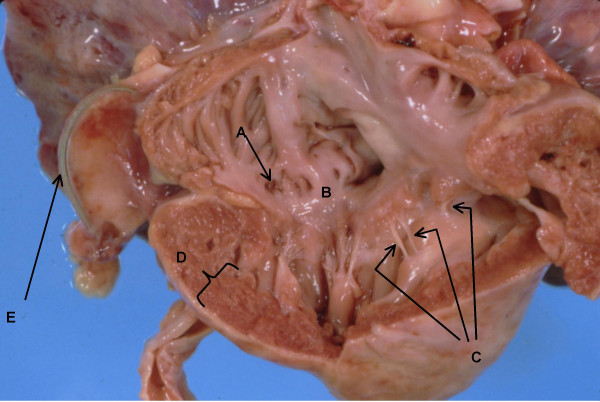
Anatomy of right ventricle and atrium. A. Dilated coronary sinus. B. Dysplastic tricuspid valve. C. Short thickened chordae tendinae almost implanted into papillary muscle. D. Right ventricular hypertrophy with ventricular wall thickness of 8 mm. E. Pacer wire.

**Figure 2:**
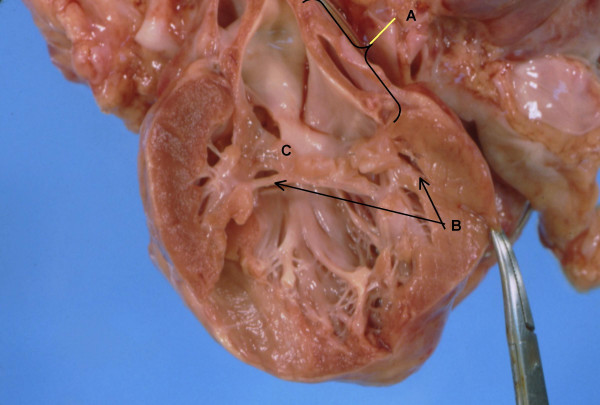
Anatomy of left ventricle and atrium. A. Left atrium with endocardial fibroelastosis. B. Short thickened chordae tendinae with direct insertion of posterior mitral valve leaflet into papillary muscle. C. Thickened dysplastic mitral valve with stenosis.

**Figure 3:**
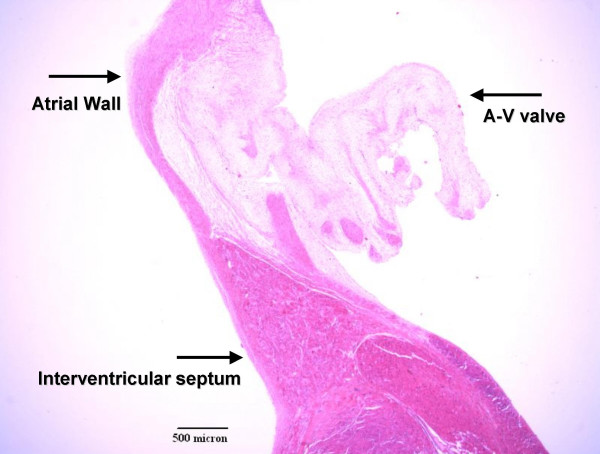
Histology of Conduction system. VVG stain.

The heart weighed twice the normal expected weight and there was biventricular hypertrophy, with the right ventricular wall thickness being about four times the normal thickness and the left ventricular wall thickness about twice normal (Figure 1). The right atrium and the coronary sinus were dilated and the left atrium was small with endocardial fibroelastosis (Figure 2). The mitral valve was stenotic, the circumference being about 3/4ths the normal circumference. The valve leaflets were thick and myxoid, and the chordae tendinae were shortened. The posterior leaflet of the mitral valve was directly inserted into the papillary muscle. There was mild aortic stenosis and the aortic valve leaflets were thick and dysplastic. The pulmonary valve circumference was about one-third greater than normal. The membranous portion of the interventricular septum was about three times the normal length and mapping of the junctional tissue revealed that the atrioventricular node was displaced caudally and the bifurcation of the Bundle of His was likewise displaced caudally (Figure 3). The valve leaflet was made up of primarily mucopolysaccharide material (blue stain). The placement of the pacemaker was verified to be appropriate and dense adhesions were present between the pacer wires and the abdominal wall and bowel loops.

**Figure 4:**
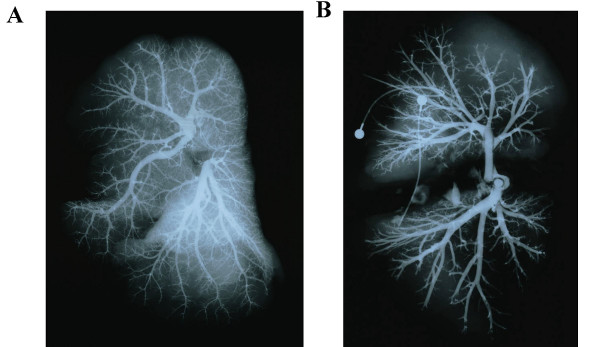
Pulmonary arteriograms. A. Age-matched normal child. B. Kabuki syndrome patient.

**Figure 5:**
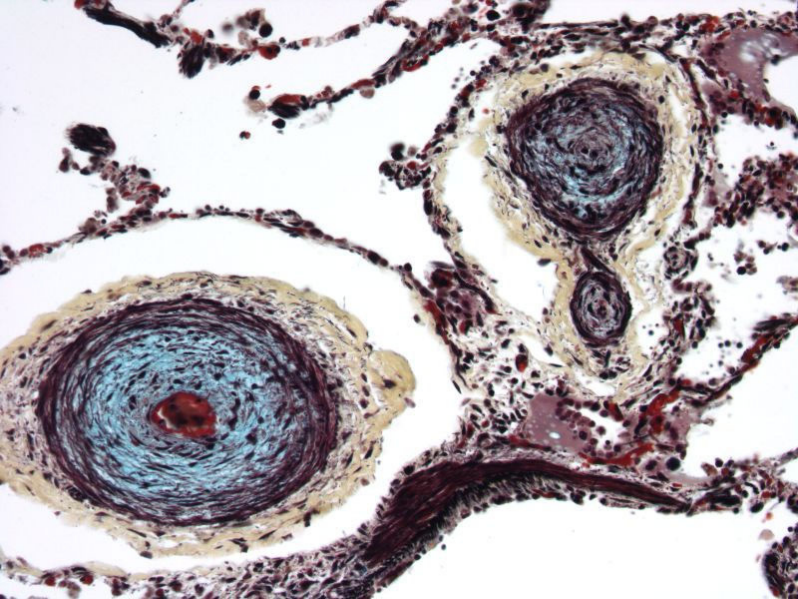
Histology of the lung. Movat pentachrome stain.

Postmortem pulmonary angiogram revealed severe pruning of the pulmonary vascular tree (Figure 4) and absence of the background 'blush' produced by filling of intracinar arteries. Microscopic examination revealed marked luminal narrowing or fibrous occlusion of intra-acinar arteries (Figure 5). In addition there was marked dilatation of lymphatics within pulmonary septa.

### Immunological phenotype

The patient exhibited recurrent infections requiring numerous hospitalizations. These included *Klebsiella *pneumonia, RSV pneumonia, *Enterococcus *sepsis, *Candida albicans *urinary tract infection, *Enterobacter *urosepsis, sinusitis and otitis media.

Immune evaluation revealed persistent lymphopenia; however, the percentages of T-, B-, and NK-cell subpopulations were normal (Table 1). Furthermore, lymphoproliferative responses to mitogens PHA, PWM and alloantigens were normal. However, lymphoproliferative response to Concavalin A stimulation was absent. Serum IgG and IgA levels were markedly decreased, and antibody responses to tetanus toxoid and *Hemophilus influenzae *type B (HiB) were decreased. Following immunization with conjugated pneumococcal vaccine (Prevnar), antibody responses were decreased to 4 of 7 serotypes. Serum albumin levels were normal, and there was no evidence of protein loss through the gastrointestinal or urinary systems, and there was no evidence of chylous thorax. Thus, IVIG therapy was initiated. However, the patient shortly succumbed from cardiac arrhythmia. Autopsy revealed severe thymic involution (the thymus weighed 1 g.) and lymphoid depletion in the spleen.

### Additional autopsy findings

Other findings included nesidioblastosis, vacuolation of the adrenal cortex, undescended testes, contraction band necrosis of the muscularis propria of the gastro-intestinal tract and growth retardation with growth parameters being < 3^rd ^percentile.

## Conclusion

Since its original description in 1981, there are now numerous reports in the literature across ethnic lines defining the phenotype of Kabuki syndrome. However, the etiology and genetics of Kabuki syndrome are poorly understood. Although a few cytogenetic abnormalities in patients have been reported including X chromosome rings [10], translocations[11], inversions[11] and duplications[12] as well as a variety of autosomal chromosomal defects[8], the majority of patients have normal chromosomes and no specific genes to date have been identified. The majority of cases are sporadic, however, a few families with multiple generations of affected persons suggests autosomal dominant inheritance [4]. In the absence of molecular diagnosis, a confirmatory diagnosis is based on clinical judgment and the reported phenotypic abnormalities associated with this syndrome have been expanded since the initial case reports. Our patient had characteristic dysmorphology, visceral abnormalities and clinical features of Kabuki syndrome. The diagnosis was determined by independent evaluation by two separate geneticists. In addition, he had cognitive delay, microcephaly, growth retardation, cleft palate, hypothyroidism, coarctation of the aorta, and diaphragmatic eventration.

Of the visceral abnormalities associated with Kabuki syndrome, congenital heart disease appears to be the most common with rates ranging from 32% to as high as 58%[2]. In regards to the specific anatomic abnormalities, there is some dispute in the literature. The earlier studies were not conclusive for specific cardiac defects while the later studies show a greater association of coarctation of the aorta. In none of these reviews or in isolated case reports has there been a report of an abnormal cardiac conduction system or reports of arrhythmia. This child had abnormalities in his cardiac conduction system that eventually lead to numerous episodes of bradycardia and eventually to asystole. Although he had structural cardiac abnormalities which can often result in alterations in chamber size and predispose to arrhythmia, his surgical correction was appropriate and there does not appear to have been secondary strain on the ventricles. A noted abnormality on evaluation of his AV node and Purkinje tracts was their altered placement. After his asystolic episode leading to death, his pacemaker was queried and showed normal functioning. During the time of bradyarrythmia it appeared to fire appropriate without normal capture. The abnormal placement of his conduction system likely contributed to the lack of pacemaker pickup.

Increased susceptibility to infections has been reported as a frequent complication in KS. Recurrent otitis media has been reported in 63% of patients with KS. This has often been attributed to anatomic reasons secondary to the cleft palate; however, this occurs in only 35% of patients with clefting of the palate not associated with a syndromic diagnosis. In addition, some of the patients have also had bacterial pneumonia and one patient had *Aspergillus fumigatus *pneumonia. Hypogammaglobulinemia with low serum IgG and IgA levels but normal IgM level has been previously reported in four patients with KS[13,14]. These patients were older than our patient when diagnosed with hypogammaglobulinemia. We believe this is the first report of an infant with KS diagnosed with hypogammaglobulinemia. Furthermore, our patient displayed the same pattern of hypogammaglobulinemia, namely hypogammaglobulinemia with normal IgM. Chrzanowska et al. [9] also diagnosed an associated T-cell defect with the hypogammaglobulinemia in a 10-year-old boy. Though our patient did have lymphopenia (1818 cells/mm^3^), percentages of T-, B- and NK-cell populations were normal. Furthermore, naïve T-cells were normal, CD3+CD45RA+, 79%. This is contrast to decreased naïve CD4+ T-cells reported by Chrzanowska [9]. Importantly, lymphoproliferative responses to PHA, PWM and alloantigens were normal. However, response to Concavalin A stimulation was absent, perhaps indicating a T suppressor defect.

Immune cytopenias have been previously reported in KS. Niikawa et al. [13] reported hemolytic anemia and Watanabe et al. [14] reported idiopathic thrombocytopenia. Autoimmune cytopenias have been associated with common variable immunodeficiency (CVID) [15] and hypogammaglobulinemia with normal IgM deficiency (hyper-IgM syndrome) [16]. The pattern of low IgG and IgA with normal IgM concentrations may be seen in both CVID and hyper-IgM syndromes. In KS, the hypogammaglobulinemia has generally been described as acquired hypogammaglobulinemia, occurring in older children. Our patient is the first description of a probable congenital diagnosis of hypogammaglobulinemia most likely from CVID.

Herein we describe a male infant with Kabuki syndrome presenting with cardiac arrhythmia and congenital immunodeficiency. Based on our single case report, we do not advocate changes in management of patients with Kabuki syndrome. However, those with cardiac abnormalities should be monitored closely during times of hospitalization for cardiac arrhythmias. Those children presenting with arrhythmias may warrant electrophysiological evaluation. Additionally, it may be beneficial for children with recurrent infections associated with a diagnosis of Kabuki syndrome to have a thorough immunologic evaluation. The association of hypogammaglobulinemia and KS supports a genetic etiology. Future studies of the hypogammaglobulinemia B-cell subsets, expression of IgG and IgA surface B-cells, and IgM to IgG isotype switching would better characterize the immunologic phenotype in this syndrome. Appropriate prevention strategies should be implemented to decrease the likelihood of a catastrophic infection.

## Competing interests

The author(s) declare that they have no competing interests.

## Authors' contributions

B.B. and M.M were responsible for chart review and organization of pertinent material and contributed to the writing and editing of the manuscript. B.B. and D.D were responsible for procurement and analysis of pathologic specimens. A.K. was a significant contributor in writing this manuscript and was responsible for the conduction and interpretation of immunologic studies. M.S. initially diagnosed this child and was responsible for the final writing, editing, organization and submission of this manuscript.

## Pre-publication history

The pre-publication history for this paper can be accessed here:



## Figures and Tables

**Table 1:** Comparison of immunophenotypes.

**Study**	**Patient**	**Normal for Age**
*Phenotype Analysis*		
Lymphocytes/mm^3^	1818	6000 ± 1500
CD2, %	70	73 ± 8
CD3, %	62	66 ± 13
CD4, %	42	43 ± 12
CD8, %,	19	25 ± 9
CD45RA+CD3, %	79	64 – 93
CD45RO+CD3, %	11	
CD25+CD4, %	14	<3
CD20, %	14	8 ± 3
smIgM, %	11	4 – 16
smIgM, %	10	3 – 15
CD56, %	10	13 ± 7
*Lymphoproliferative Responses*		
PHA, cpm	164,082	100,530 – 657,376
%NR	65	>50
Con A, cpm	10	53,173 – 502,758
%NR	0	>50
PWM, cpm	121,168	40,305 – 337,597
%NR	96	>50
MLC, cpm	118,219	43,801 – 328,175
SI	23.2	>3.0
*Immunoglobulins*		
IgG, mg/dl	113	399–1068
IgA, mg/dl	11	15–95
IgM, mg/dl	97	49–202
IgE, IU/ml	<2	3–29
anti-HiB, μg/ml	<0.5	>1.0
anti-Diphtheria toxoid, IU/ml	1.61	>0.05
anti-Tetanus toxoid, IU/ml	0.2	>0.5
anti-Streptococcus, μg/ml		
Serotype 4	4.6	>2.0
Serotype 6	2.2	>2.0
Serotype 9	0.3	>2.0
Serotype 14	1.0	>2.0
Serotype 18	12.2	>2.0
Serotype 19	0.9	>2.0
Serotype 23	0.3	>2.0
CH50, U/ml	64	31 – 64

Immunological studies in our 10 month old child. PHA, phytohemagglutinin; Con A, concanavalin A; PWM, pokeweed mitogen; MLC, mixed lymphocyte culture to B-cell alloantigens; %NR, percent normal response; SI, stimulation index; HiB, *Hemophilus influenzae *type B.
